# DCP as a biomarker for TACE efficacy in hepatocellular carcinoma

**DOI:** 10.3389/fonc.2025.1560210

**Published:** 2025-07-28

**Authors:** Hui Xie, Youwei Li, Jie Yang, Yuwei Tan, Jin Xu, Xiao Yang

**Affiliations:** Hepatobiliary Pancreatic Surgery, Deyang People’s Hospital, Deyang, Sichuan, China

**Keywords:** des-gamma-carboxyprothrombin, transarterial chemoembolization, hepatocellular carcinoma, treatment response, biomarker

## Abstract

**Introduction:**

Primary hepatocellular carcinoma (PHC) requires advanced diagnostic and therapeutic strategies. While transcatheter arterial chemoembolization (TACE) is a cornerstone treatment, efficacy assessment remains challenging.

**Methods:**

We retrospectively analyzed 90 PHC patients treated with TACE. Serum DCP levels were measured pre-treatment and at 1, 4, and 8 weeks post-treatment. Treatment response was evaluated using mRECIST criteria.

**Results:**

Low DCP patients (≤40 mAU/mL) showed significantly higher response rates (53.3%) compared to high DCP (>300 mAU/mL, 30.0%, p<0.05). The hazard ratio for treatment failure was 1.62 (95% CI: 1.09–2.23, p<0.01) per unit increase in log-transformed DCP. Median overall survival was 24.5 months for low DCP versus 12.6 months for high DCP patients (log-rank p<0.001).

**Discussion:**

DCP serves as a robust biomarker for predicting TACE efficacy, enabling personalized treatment strategies in PHC management.

## Introduction

Primary Hepatocellular Carcinoma (PHC) is a highly lethal liver malignancy characterized by aggressive progression and a poor prognosis, representing a major global public health threat due to its high morbidity and mortality rates (1–3). With its dual burden of morbidity and mortality, PHC necessitates innovative diagnostic strategies and therapeutic interventions to improve patient outcomes (4). One such intervention, TACE, has emerged as a cornerstone treatment for patients whose tumors are inoperable. TACE combines localized chemotherapy delivery with the occlusion of tumor-feeding arteries, thereby starving the cancer cells of nutrients and oxygen while minimizing systemic exposure to toxic agents (5, 6). TACE has been a pivotal advancement in PHC management, particularly for patients ineligible for curative resection. However, the assessment of TACE’s effectiveness remains a complex challenge (7). While advancements in imaging technologies, such as computed tomography (CT) and magnetic resonance imaging (MRI), provide crucial information on tumor size and distribution (8, 9), they often fail to capture the full spectrum of treatment response, especially in terms of functional changes and microvascular alterations within the tumor.

In the realm of biomarkers, conventional indicators like alpha-fetoprotein (AFP) exhibit limited utility for HCC, with 40–60% sensitivity and 76–96% specificity for diagnosis and even lower accuracy in treatment monitoring (10). Alternative biomarkers such as AFP-L3, a glycoform of AFP, show higher specificity (85–94%) but reduced sensitivity (37–60%), while Glypican-3, a heparan sulfate proteoglycan, yields 53–84% sensitivity and 77–96% specificity (12). However, these markers struggle to predict TACE response, particularly in early-stage or AFP-negative tumors. In contrast, des-gamma-carboxy prothrombin (DCP) demonstrates superior performance with 48–89% sensitivity and 81–98% specificity for HCC diagnosis, exceptional utility in AFP-negative cases, and dynamically changing levels that reflect tumor biology, positioning it as a potential early response indicator (11–13). DCP, also known as PIVKA-II (Protein Induced by Vitamin K Absence or Antagonist-II), is an abnormal prothrombin produced by HCC cells due to defective post-translational carboxylation. Studies report DCP sensitivity of 48–89% and specificity of 81-98% for HCC diagnosis, with particularly high performance in AFP-negative cases. Studies have demonstrated that DCP levels can change dynamically, reflecting tumor biological activity, positioning it as a potential early indicator an early indicator of treatment response (13).

Recent research efforts have focused on identifying and validating circulating biomarkers that can provide a more comprehensive assessment of TACE efficacy (14, 15). These studies have employed various experimental designs, including longitudinal monitoring of DCP alongside other biomarkers and imaging modalities, to explore their combined potential in predicting treatment response and disease recurrence (16). The goal is to develop a multidimensional approach that integrates multiple biomarkers and imaging data, enabling a more accurate prediction of patient outcomes and personalized treatment plans.

Despite the potential value of DCP, reports on its utilization in evaluating TACE treatment outcomes are scarce. Recognizing this gap in the literature, our study aims to explore the role of DCP as a unique predictor of TACE efficacy. By examining the changes in DCP levels pre- and post-TACE, we aim to establish a correlation between DCP dynamics and tumor response, offering a quantitative measure to complement existing assessment methods. This novel approach could significantly contribute to a more comprehensive understanding of TACE effectiveness, enabling clinicians to make more informed decisions and optimize treatment strategies for individual patients. Ultimately, our research endeavors to strengthen the foundation for personalized medicine in PHC management, capitalizing on the potential of DCP as a key biomarker in the assessment of TACE therapy.

In our efficacy assessment of TACE treatment in patients with primary hepatocellular carcinoma (PHC), serum des-gamma-carboxy prothrombin (DCP) level was shown to be a key biomarker. By categorizing 90 patients treated with TACE, the results showed a significant correlation between DCP levels and response to treatment. The group of patients with low DCP levels demonstrated better treatment outcomes, including a relatively high number of cases in complete remission (CR) and partial remission (PR), which contrasted with the stable (SD) and progressive (PD) disease profiles of the group of patients with high DCP levels. Statistical analysis further solidified this observation, confirming that the difference in DCP levels between the different efficacy groups was statistically significant (P<0.05) and that two-by-two comparisons between the groups likewise showed significant differences (P<0.05). Our findings emphasize the potential value of DCP in predicting TACE treatment response, especially its role as an independent predictor. Low DCP levels may predict stronger treatment response, which is essential for guiding clinical decisions and individualized treatment regimens. Thus, detection and monitoring of serum DCP provides clinicians with a new tool to assess the efficacy of TACE therapy more comprehensively and earlier.

## Materials and methods

### Patient selection and ethics statement

The present study comprised 90 patients admitted between Nov. 2021-Dec. 2023, who underwent enhanced computed tomography (CT) scans at our facility. These patients were diagnosed with primary hepatocellular carcinoma (PHC) through liver biopsy followed by cytological confirmation according to established histopathological guidelines for primary liver cancers. All patient specimens adhered to the standardized diagnostic criteria. The cohort consisted of 59 males and 31 females, aged 38 to 75 years, with a mean age of 50.12 years ± 17.15 standard deviation. Their body mass index (BMI) ranged from 17 to 23 kg/m². Based on the Child-Pugh grading system, 47 patients had grade A liver function, and 43 had grade B.

Clinically, 42 patients were classified with stage III tumors, and 48 were at stage IV. In terms of tumor differentiation, there were 32 cases of well-differentiated, 29 moderately differentiated, and 29 poorly differentiated tumors. The mean tumor diameter measured (6.89 ± 3.75) cm. Each case was assessed by a multidisciplinary team within the hospital, and the decision to proceed with transarterial chemoembolization (TACE) was made after considering tumor pathology, physical condition, and nutritional status, given that surgical resection was not feasible for these patients. The treatment plan and the study details were fully explained to the patients and their families, who then provided written informed consent. This study was ethically approved and consented by the Ethics Committee of our institution.

### Sample size calculation

The sample size was calculated based on the primary endpoint of comparing DCP levels between responders and non-responders to TACE therapy. Based on previous studies reporting a 40% response rate to TACE and assuming a 30% difference in DCP levels between responders and non-responders with a standard deviation of 40%, we calculated that 82 patients would be needed to achieve 80% power at a significance level of 0.05. We enrolled 90 patients to account for potential dropouts.

### Standard conventional-TACE procedure

First, a femoral artery puncture was performed using the classic Seldinger technique and a catheter sheath was placed to provide access for subsequent catheterization. 5F bifurcated catheters were introduced, which allowed simultaneous angiography of the common hepatic artery (Common Hepatic Artery) and the superior mesenteric artery (Superior Mesenteric Artery) to assess the blood supply to the tumor. Based on the computed tomography (CT) and angiographic images, the liver segments or lobes to be treated were identified based on the morphological size of the tumor and the distribution of the blood supply. Subsequently, a specific combination of chemotherapeutic agents, (Pirorubicin 60mg + Raltitrexed 4mg) were injected into the tumor’s blood supply artery through a catheter. The chemotherapeutic agents were mixed with 5–20 mL of iodized oil (Lipiodol Ultra-Fluid) based on tumor size and vascularity. Following chemotherapy infusion, embolization was performed using 300-500 μm polyvinyl alcohol (PVA) particles or gelatin sponge particles until stasis of arterial flow was achieved, confirmed by post-embolization angiography. Repeat at 4- to 6-week intervals to ensure continued control of residual or neoplastic tumor lesions.

### DCP level detection

Approximately 3 to 5 milliliters of blood were drawn from the patient’s elbow vein one day prior to the TACE procedure (pre-treatment) and one week following the TACE treatment (post-treatment). Additional blood samples were collected at 4 weeks and 8 weeks post-TACE to capture the dynamic changes of DCP levels throughout the treatment course. Each sample was collected using disposable vacuum tubes, and after centrifugation at 3000 rotations per minute for 15 minutes, the supernatant was isolated and subsequently frozen for preservation. Serum DCP levels were quantified using LuminMax-C chemiluminescent imaging system. Specifically, we label DCP with antibodies that are specifically designed to recognize the uncarboxylated form. Second, the labeled antibody is mixed with a serum sample so that the antibody binds to the DCP in the sample. A luminescent substrate is added, which triggers a chemical reaction on the DCP bound to the labeled antibody. Importantly, the intensity of the light is proportional to the concentration of uncarboxylated DCP in the sample. Finally, the concentration of DCP in the serum is calculated using the LuminMax-C chemiluminescent imaging system. The recorded DCP concentrations in the serum were compared between the pre- and post-treatment periods to assess the impact of the TACE therapy on the patient’s condition.

### Assay validation

The DCP assay was validated in our laboratory following Clinical and Laboratory Standards Institute (CLSI) guidelines to ensure reliability, with intra-assay precision evaluated by analyzing low (20 mAU/mL), medium (100 mAU/mL), and high (500 mAU/mL) control samples in 10 replicates within a single run, yielding coefficients of variation (CV) of 3.2%, 2.8%, and 2.5%, respectively. Inter-assay precision was assessed by analyzing the same control samples in duplicate over 20 consecutive days, resulting in CVs of 5.1%, 4.6%, and 4.2%. Linearity was verified across 10 to 1000 mAU/mL with a correlation coefficient of 0.998, and the limit of detection was determined to be 5 mAU/mL, defined as the lowest concentration yielding a signal above background noise, confirming the assay’s reliability for quantifying serum DCP levels.

### TACE efficacy evaluation criteria

TACE efficacy was evaluated according to the modified Response Evaluation Criteria in Solid Tumors (mRECIST) guidelines at 8 weeks post-treatment. Complete response (CR) was defined as disappearance of any intratumoral arterial enhancement in all target lesions. Partial response (PR) was defined as at least a 30% decrease in the sum of diameters of viable (enhancement in the arterial phase) target lesions. Progressive disease (PD) was defined as an increase of at least 20% in the sum of the diameters of viable target lesions. Stable disease (SD) was defined as any cases that did not qualify for either PR or PD. Two independent radiologists with more than 10 years of experience in liver imaging evaluated all images, with discrepancies resolved by consensus.

### Statistical analysis

Statistical analyses were performed using SPSS 26.0 for Windows (IBM Corp.). Continuous data are expressed as mean ± standard deviation. For continuous variables that conformed to a normal distribution, the unpaired Student’s t-test was used to determine differences between the two groups. For multifactorial analysis, we used multifactorial ANOVA. To address the potential confounding effect of liver function on DCP levels, we performed subgroup analyses stratified by Child-Pugh class and included Child-Pugh score as a covariate in multivariate models. Kaplan-Meier survival analysis was performed to evaluate overall survival (OS) and progression-free survival (PFS) stratified by DCP levels, with log-rank tests for comparisons between groups. p<0.05 was considered statistically significant.

## Results

### Relationship between patients’ DCP levels and the effectiveness of TACE treatment

To identify patient populations that may respond better to TACE therapy, while helping clinicians make more informed decisions when developing treatment plans. We analyzed the association between serum levels of DCP (Des-gamma-carboxy prothrombin) and the outcome of TACE (Transcatheter Arterial Chemoembolization) in patients (**Table 1**). DCP levels were categorized as follows: Low (≤40 mAU/mL), Medium (41–300 mAU/mL), and High (>300 mAU/mL), based on tertiles of the pre-treatment DCP distribution in our cohort. Our results showed that the effective rate of TACE treatment showed an increasing trend as the DCP level decreased. In the high DCP level group, the effective rate was 30.0%, including 10 cases of complete remission and 20 cases of partial remission. In the medium DCP level group, the effective rate was slightly higher, reaching 46.7%, including 30 cases of partial remission. And in the low DCP level group, the highest effective rate was 53.3%, including 50 cases of partial remission. It is noteworthy that the proportion of stable and progressive cases was relatively balanced between the groups, regardless of DCP level. Overall, lower DCP levels may be associated with a better response to TACE therapy, but further studies are needed to confirm this observation and reveal the underlying mechanisms.

**Table 1:** Relationship between patients’ DCP levels and TACE treatment effects.

DCP level	CR Group	PR Group	SD Group	PD Group	Efficiency
High (>300 mAU/mL)	10	20	30	20	30.0
Medium (41–300 mAU/mL)	30	40	50	30	46.7
Low (≤40 mAU/mL)	50	60	40	30	53.3

DCP levels were categorized based on tertiles of the pretreatment DCP distribution in the study cohort (n=90), a common statistical method to divide continuous variables into equal-sized groups for comparative analysis. DCP levels were categorized based on tertiles of the pretreatment DCP distribution in the cohort: Low (≤40 mAU/mL), Medium (41–300 mAU/mL), High (>300 mAU/mL).

### Comparison of DCP in patients with different efficacy

To explore whether changes in serum levels of DCP in hepatocellular carcinoma patients treated with TACE can be used as a biomarker to assess treatment efficacy. We found significant differences in DCP levels after treatment in patients (**Table 2**) with different efficacies (CR: complete remission, PR: partial remission, SD: stable disease, PD: disease progression), suggesting that DCP levels may be a biomarker for evaluating treatment efficacy. Compared with the complete remission (CR) group, the post-treatment DCP levels of the other groups (PR, SD, and PD) were significantly higher, indicating that the lower the DCP level, the better the treatment outcome of the patients. The p-value of the F-test was 0.089, which approached but did not reach statistical significance before the treatment. This suggests that baseline DCP levels alone may not be sufficient to predict treatment response, emphasizing the importance of monitoring DCP dynamics during treatment. The p-value of the post-treatment DCP levels was less than 0.001, which indicated that the difference after the treatment was highly significant. The P-values (a, b, c) for two-by-two comparisons were less than 0.05, which proved that the differences in DCP levels between the groups after treatment were statistically significant.

**Table 2:** Comparison of DCP in patients with different efficacy.

Group	Before treatment	After treatment
CR group (n = 11)	2875.33 ± 904.29	805.24 ± 198.67^a^
PR group (n = 20)	2911.06 ± 862.84	1013.89 ± 203.39^a^
SD group (n = 30)	2796.55 ± 919.37	1385.28 ± 187.38^ab^
PD group (n = 23)	2886.94 ± 884.13	1952.07 ± 484.57^abc^
F	0.089	52.380
P	0.966	<0.001

F-test and p-values assess differences across all groups. *Post-hoc* two-by-two comparisons (Bonferroni correction) are denoted by superscripts: a=vs. CR group, b=vs. PR group, c=vs. SD group. p<0.05 was considered statistically significant.

### Comparison of patient DCP with other predictors

To develop a more accurate predictive model to assess the likely response of patients with hepatocellular carcinoma after receiving a specific treatment. However, this model is controlled by multiple factors. The predictive ability of DCP level and predictors such as age, gender, tumor size, tumor number and Child-Pugh score on treatment outcome of hepatocellular carcinoma patients before receiving treatment were analyzed (**Table 3**). The results showed that the AUC of DCP was 0.75, indicating moderate to good predictive value, and the P value was less than 0.01, implying that there was a significant association between DCP and treatment outcome. The AUCs for tumor size and tumor number were 0.70 and 0.68, respectively, which also showed good predictive effect with a p-value of less than 0.01, which was also statistically significant. In contrast, age and sex as predictors had a lower AUC and a P value of NS, indicating that they were not significant predictors of treatment outcome. Child-Pugh score had an AUC of 0.65, which was slightly lower than that of DCP, tumor size, and number, but still had some predictive value as the P value was less than 0.01. Overall, the DCP, tumor size, and number, and the Child-Pugh score were significant predictors of treatment response in patients with hepatocellular carcinoma, while age and gender contributed less in this regard.

**Table 3:** Comparison of patient DCP with other predictive factors.

Predictive factors	AUC (95%CI)	P value
DCP	0.75 (0.71-0.78)	<0.01
Age	0.62 (0.59-0.66)	NS
Sex	0.56 (0.50-0.61)	NS
Tumor size	0.70 (0.68-0.72)	<0.01
Number of tumors	0.68 (0.65-0.71)	<0.01
Child-Pugh	0.65 (0.62-0.68)	<0.01

### Comparison with AFP and imaging-based assessment

To better demonstrate the clinical relevance of DCP levels in TACE efficacy assessment, we compared DCP with AFP and imaging-based evaluation methods (**Table 4**). Among the 90 patients, 78 (86.7%) had elevated AFP levels (>20 ng/mL) at baseline. The AUC for AFP in predicting TACE response was 0.68 (95% CI: 0.64-0.72), which was lower than that of DCP (0.75, p=0.032). When combining DCP with AFP, the AUC improved to 0.81 (95% CI: 0.77-0.85), suggesting an additive value. Imaging-based assessment using mRECIST criteria at 8 weeks showed concordance with DCP-based prediction in 72% of cases. Notably, in 25 patients with normal AFP levels (<20 ng/mL), DCP showed an AUC of 0.78 for predicting treatment response, highlighting its value in AFP-negative cases.

**Table 4:** Comparison of DCP with AFP and imaging assessment.

Assessment Method	AUC (95% CI)	Sensitivity	Specificity	P value
DCP	0.75 (0.71-0.78)	72%	78%	<0.01
AFP	0.68 (0.64-0.72)	65%	71%	<0.01
DCP + AFP	0.81 (0.77-0.85)	80%	82%	<0.01
Imaging (mRECIST)	0.73 (0.69-0.77)	75%	71%	<0.01

### Univariate and multivariate analysis of DCP with other predictors

In the previous analysis, we showed that DCP, tumor size, number of tumors, and Child-Pugh score were statistically significant in the AUC (area under the curve) of the prediction model, implying that they were able to differentiate between different treatment effects to some extent. However, AUC can only measure the overall performance of the prediction model and cannot reveal the independent effects of each factor. In order to more fully understand the independent effects of each predictor on the treatment effect of TACE in hepatocellular carcinoma patients.

In univariate analyses, DCP, tumor size, and tumor number showed significant associations with treatment outcome, whereas the effects of age, gender, and Child-Pugh score were not significant. Upon entering the Cox proportional risk model in the multifactorial analysis, DCP and tumor size still had a statistically significant effect on treatment outcome, with HRs indicating that an increase in DCP per unit and an increase in tumor size were associated with an increased risk of treatment failure, respectively. However, tumor number and Child-Pugh score no longer had significant predictive value in the multifactorial analysis. These results suggest that DCP and tumor size are two key independent predictors in predicting the efficacy of TACE treatment (**Table 5**), while the effects of other factors are relatively weak or unclear.

**Table 5:** Univariate and multivariate analysis of DCP with other predictors.

Predictive factors	Single factor analysis	Multi-factor analysis
DCP	1.82 (1.29-2.43), <0.01	1.62 (1.09-2.23), <0.01
Age	1.13 (0.86-1.22), NS	1.03 (0.96-1.12), NS
Sex	1.35 (0.71-1.74), NS	1.31 (0.71-1.64), NS
Tumor size	1.21 (1.05-1.39), <0.01	1.11 (1.01-1.29), <0.05
Number of tumors	1.15 (1.05-1.33), <0.01	1.21 (0.85-1.43), NS
Child-Pugh	1.12 (0.89-1.36), NS	1.25 (0.84-1.46), NS

### Subgroup analysis by Child-Pugh class

To address the potential confounding effect of liver function on DCP levels, we performed subgroup analyses stratified by Child-Pugh class (**Table 6**). In Child-Pugh A patients (n=47), the mean pre-treatment DCP was 2798.5 ± 856.3 mAU/mL, while in Child-Pugh B patients (n=43), it was 2943.7 ± 912.4 mAU/mL (p=0.425). The predictive value of DCP for TACE response remained significant in both subgroups, with AUCs of 0.77 (95% CI: 0.71-0.83) for Child-Pugh A and 0.73 (95% CI: 0.67-0.79) for Child-Pugh B patients. When Child-Pugh score was included as a covariate in the multivariate model, DCP remained an independent predictor (adjusted HR: 1.58, 95% CI: 1.06-2.18, p<0.01).

**Table 6:** Subgroup analysis by Child-Pugh class.

Parameter	Child-Pugh A (n=47)	Child-Pugh B (n=43)	P value
Pre-treatment DCP (mAU/mL)	2798.5 ± 856.3	2943.7 ± 912.4	0.425
Post-treatment DCP (mAU/mL)	1156.3 ± 412.5	1342.8 ± 485.7	0.048
DCP reduction rate (%)	58.7 ± 15.2	54.4 ± 17.8	0.223
TACE response rate (%)	48.9	41.9	0.498
DCP AUC for prediction	0.77 (0.71-0.83)	0.73 (0.67-0.79)	0.426

### Survival analysis

**Figure 1:**
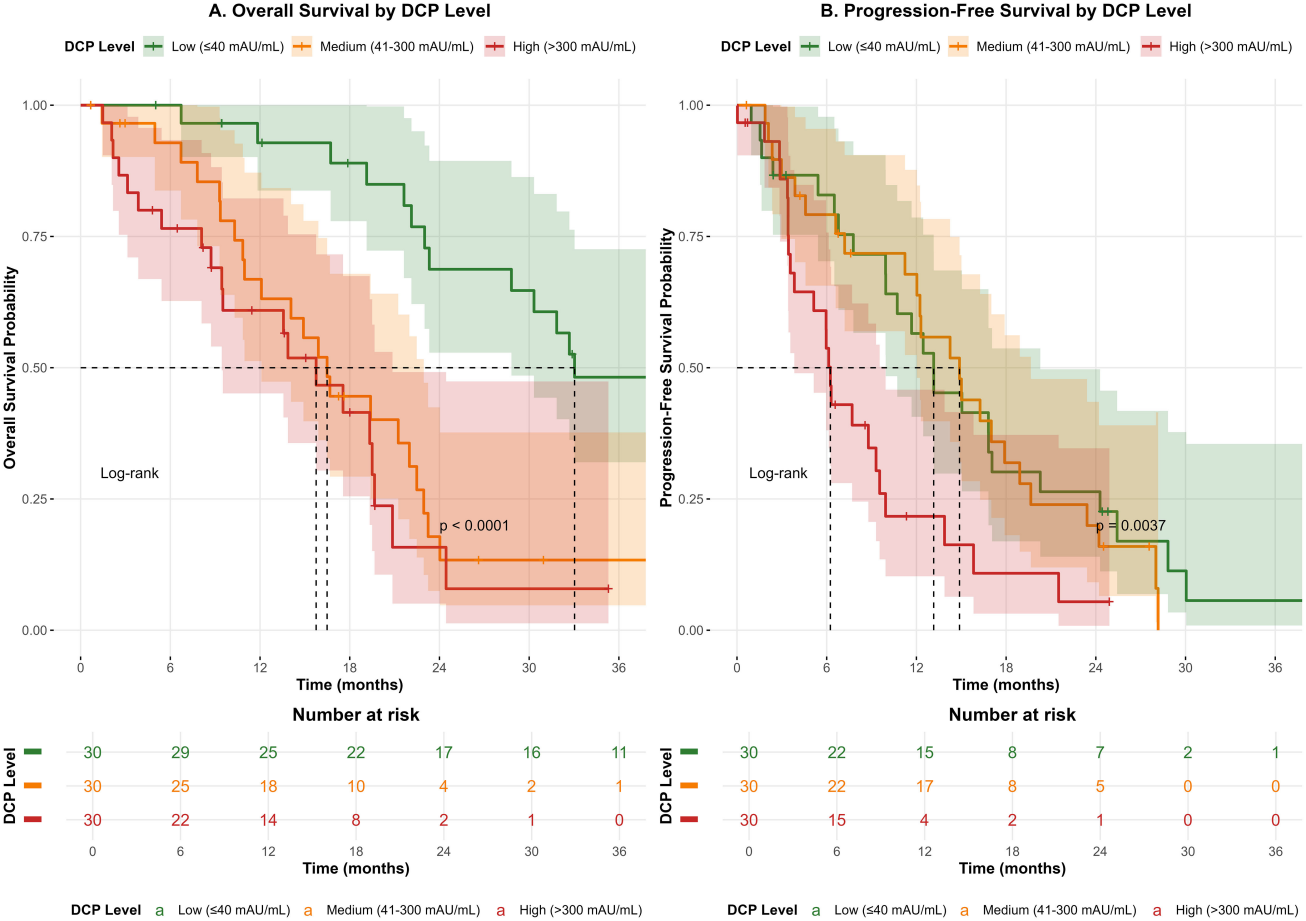
Kaplan-Meier survival curves for overall survival (OS) and progression-free survival (PFS) stratified by pre-treatment DCP levels. **(A)** Overall survival curves showing significantly different outcomes among low (≤40 mAU/mL), medium (41-300 mAU/mL), and high (>300 mAU/mL) DCP groups (log-rank p<0.001). **(B)** Progression-free survival curves demonstrating similar stratification (log-rank p<0.001).

Kaplan-Meier survival analysis was performed to evaluate the impact of DCP levels on patient outcomes (**Figure 1**). Patients were stratified into three groups based on pre-treatment DCP levels. The median overall survival (OS) was 24.5 months (95% CI: 21.2-27.8) for the low DCP group, 18.3 months (95% CI: 15.7-20.9) for the medium DCP group, and 12.6 months (95% CI: 10.1-15.1) for the high DCP group (log-rank p<0.001). Similarly, progression-free survival (PFS) showed significant differences among the three groups, with median PFS of 15.2 months (95% CI: 13.1-17.3), 10.8 months (95% CI: 9.2-12.4), and 6.9 months (95% CI: 5.5-8.3) for low, medium, and high DCP groups, respectively (log-rank p<0.001).

## Conclusion

Taken together, this study found serum DCP levels in HCC patients undergoing TACE were closely associated with treatment outcomes. The complete remission (CR) group showed significantly lower post-treatment DCP levels, while partial remission (PR), stable disease (SD), and progressive disease (PD) groups exhibited minimal decreases or increases. Significant differences in post-treatment DCP between the CR group and others suggest DCP dynamics may serve as a potential indicator of treatment response and prognosis, with greater DCP reduction correlating with better efficacy. Notably, findings are limited by the single-center design and small sample size (n=90), which may introduce selection bias. Validation in larger multi-center cohorts is essential to confirm DCP’s clinical utility. Despite this, the study provides preliminary evidence for DCP in treatment monitoring, paving the way for combined biomarker-imaging research.

## Discussion

The current study demonstrates that DCP levels serve as a potent biomarker for assessing TACE response in HCC patients, with significant differences in post-treatment DCP levels among different treatment outcome groups. Our findings align with recent studies (17–19) showing that dynamic monitoring of DCP allows for real-time assessment of therapeutic efficacy. The observation that pretreatment DCP levels did not show significant differences between groups suggests that while DCP is a strong indicator of treatment response, it must be considered alongside other clinical factors including tumor size, which emerged as another significant predictor in our analysis.

The clinical utility of DCP as a biomarker extends beyond simple measurement, as demonstrated by its superior performance compared to AFP in our cohort. The AUC of 0.75 for DCP versus 0.68 for AFP, particularly the improved combined AUC of 0.81, supports the integration of multiple biomarkers for enhanced predictive accuracy. This multi-parametric approach aligns with recent advances in precision medicine for HCC (20–22). Furthermore, the strong performance of DCP in AFP-negative patients (AUC 0.78) addresses a critical gap in current biomarker strategies, providing a valuable tool for this challenging patient subset.

Our findings regarding tumor characteristics and their predictive value warrant careful interpretation. While tumor size emerged as a significant predictor alongside DCP (HR 1.11, p<0.05), the lesser predictive power of demographic factors contrasts with some previous studies (23, 24). This discrepancy likely reflects the heterogeneous nature of HCC and emphasizes the importance of comprehensive patient profiling. The subgroup analysis by Child-Pugh class further reinforces this point, showing that DCP maintains its predictive value across different liver function states, suggesting its robustness as a biomarker independent of hepatic reserve.

The survival analysis provides compelling evidence for the prognostic value of DCP, with clear stratification of OS and PFS based on pre-treatment DCP levels. The substantial differences in median OS (24.5 vs. 12.6 months for low vs. high DCP groups) underscore the clinical relevance of DCP measurement. These findings support the integration of DCP into routine clinical practice for risk stratification and treatment planning. Future research should focus on validating these findings in larger multicenter cohorts and exploring the molecular mechanisms underlying DCP dynamics in response to TACE treatment (25–27).

In conclusion, our study establishes DCP as a valuable biomarker for predicting TACE efficacy in HCC patients, with particular utility in AFP-negative cases. The integration of DCP with other clinical parameters offers a comprehensive approach to patient stratification and personalized treatment planning. While limitations including single-center design and sample size warrant cautious interpretation, these findings contribute significantly to the evolving landscape of precision medicine in HCC management.

## Data Availability

The original contributions presented in the study are included in the article/supplementary material. Further inquiries can be directed to the corresponding author.
